# Does the use of universal adhesive systems improve the durability of the bond strength of orthodontic brackets to enamel?

**DOI:** 10.4317/jced.61247

**Published:** 2024-02-01

**Authors:** Isabella-Saraiva-Leão de Resende, Diana-Araújo Cunha, Paulo-Goberlânio-de Barros Silva, Juliana-Ximenes Damasceno, Lara-Rabelo Aragão, Anya-Pimentel-Gomes-Fernandes Vieira-Meyer, Jiovanne-Rabelo Neri

**Affiliations:** 1 Master of Dental Sciences, University Center Christus (Fortaleza, Ceará, Brazil)

## Abstract

**Background:**

Universal adhesive systems used for restorative clinical procedures are like orthodontics and may be a viable option. This study evaluated the effectiveness of universal adhesive systems in enhancing the durability of the shear bond strength (SBS) and adhesive remnant index (ARI) of orthodontic brackets to enamel.

**Material and Methods:**

100 bovine incisors were divided into five groups (n=20), according to the applied adhesive systems: Primer Transbond XT; Ambar; Ambar Universal; Single Bond Universal; Adper Single Bond 2. Bracket from each tooth were submitted to SBS test after 24 hours, and 12 months later. The amount of remaining adhesive was evaluated through ARI.

**Results:**

After 24 hours, there was no difference in BS between the control and the other groups (p>0.05). However, there were difference between TOTALETCHING1 group and the Ambar Universal (*p*=0.015) and Single Bond Universal groups (*p*=0.011). After 12 months, Primer Transbond XT, Ambar, Ambar Universal and Adper Single Bond 2 showed no differences in the SBS (*p*>0.05). Nonetheless, Single Bond Universal presented superior result when compared to Primer Transbond XT (*p*=0.046) and Ambar (*p*=0.011) groups. The SBS of all groups reduced significantly after 12 months (*p*<0.05). There was no difference between ARI scores in each individually assessed group (*p*>0.05), for both periods. Following 24 hours, a difference was observed between the groups (*p*=0.043), fact that didn’t occur after 12 months (*p*=0.109).

**Conclusions:**

Adhesive systems, such as Ambar Universal and Single Bond Universal are efficient in bonding orthodontic brackets to enamel when associated with Transbond XT adhesive paste.

** Key words:**Bond strength; Primer Transbond XT, orthodontic brackets, adhesive systems.

## Introduction

The development of the enamel acid-etching technique by Buonocore (1) enabled a substantial advance in Orthodontics, with the possibility of replacing orthodontic bands by the direct bonding of accessories, such as brackets, tubes, and buttons, to the enamel (2). Success in Orthodontics therapy is directly related to the effectiveness of the bond promoted by adhesive systems, which must be capable of resisting masticatory and Orthodontics forces, avoiding the accidental detachment of accessories (3). Although numerous studies have addressed the issue during the last two decades (4-7), orthodontics, mainly due to the diversity of products available on the market, still having questions regarding the choice of adhesive system to utilize in their clinical practice (8).

Tooth enamel is a homogeneous substrate composed of 96% inorganic matter, which renders adhesive procedures in this tissue predictable, mainly when associated with the conventional enamel acid-etching technique by phosphoric acid (9). However, the use of more simplified methods, with fewer clinical steps, became the target of studies in Orthodontics (2,7,10). The application of self-etching and universal adhesives in the bonding of Orthodontics brackets has been encouraged, despite the uncertainty regarding its bond effectiveness/longevity (4,11), which can be seen by the variation of results found in the literature (12,13).

Considering that Orthodontics treatments take months, or years until finalized, a factor that deserves emphasis is the durability of the bond promoted by the adhesive systems. This aspect is little considered by adhesion studies in Orthodontics, and, in some cases, its evaluation is only assessed during short periods, which does not correspond to the reality of treatment with braces (6,7).

Adhesive systems have a substantial influence on the efficiency of Orthodontics treatment, thus accounting for the requirement of their constant evaluation. Adhesive systems specifically indicated for bonding Orthodontics brackets are now available on the market, some of which have already been recognized in the literature regarding their effectiveness (2,11,14). Several studies have been carried out to investigate the quality of the bond promoted by adhesive systems (6,7,14), but not all of them evaluate the use of non-specific adhesives for Orthodontics (3,12,15). Additionally, little is known about the durability of the bond established between Orthodontics accessories and adhesive systems indicated for restorative procedures.

The possibility of using non-specific adhesive systems for Orthodontics on the bonding of orthodontic brackets may lead to the simplification of dental practice and reduction of costs with materials, since the clinician may employ the same adhesive system for different procedures, either restorative or orthodontic. We also consider that the search for adhesive systems that combine long-term efficacy, cost-effectiveness, and multiple functionalities is relevant. Thus, the aim of this study was to evaluate the shear bond strength (SBS) and the adhesive remnant index (ARI) of orthodontic brackets bonded to enamel using different adhesive systems (e.g., restorative, or orthodontic). The null hypothesis was that there would be no statistical difference between SBS and ARI, regardless of the adhesive system applied and the storage period.

## Material and Methods

This *in vitro* experimental study was performed under the approval of Ethics Committee for Animal Research (protocol 003/18). One hundred extracted bovine incisors, lacking caries, cracks, or fractures on the buccal surface, were selected, donated from a public slaughterhouse. The teeth were cleaned using periodontal curettes (Gracey - Golgran, São Caetano do Sul, SP, Brazil) for the complete removal of the periodontal ligament and maintained in distilled water at 4°C until the experiments were conducted. The storage solution was changed every 15 days to avoid bacterial proliferation.

The bovine incisors underwent root sectioning 10 mm below the amelo-cementum limit using a diamond saw in a metallographic cutter (60 rpm, Biopdi, São Carlos, SP, Brazil), and the apical ends of the roots were discarded. Planification of the buccal surfaces were performed using #320-grit and #600-grit silicon carbide paper mounted to an electric polishing machine (Aropol-2V; Arotec Indústria e Comércio, Cotia, SP, Brazil), under abundant irrigation. The teeth were then fixed with self-curing acrylic resin (VIPI, Pirassununga, SP, Brazil - Lot# 0000077672), in PVC cylinders (Tigre, São Paulo, SP, Brazil) measuring 25 mm in diameter by 25 mm in height, in a way that the buccal surfaces were perpendicular to the base of the PVC ring, and the root remnants were immersed in the acrylic resin. An acrylic square was used to standardize the positioning of the teeth on the PVC ring, ensuring that the buccal surfaces were parallel to the force application direction in the shear tests. Next, prophylaxis of the buccal surfaces was conducted using pumice stone paste (SS White, Rio de Janeiro, RJ, Brazil) and water, applied in a rubber cup (Microdont, Monsey, NY, USA), followed by rinsing and drying with a triple syringe.

The specimens were randomly divided (Excel 2013, Microsoft Corporation, Redmond, WA, USA) into 5 equal groups (n=20) according to the adhesive system applied (Table 1,  Table 1 cont.). In all groups, Transbond XT orthodontic composite (3M ESPE, St. Paul, MN, USA - Lot# N841326) was used for bonding the brackets (Stainless steel, Roth Standard for lower incisors/ Morelli, Sorocaba, SP, Brazil - Lot# 2316573). The bonding was performed using manual pressure, by a single calibrated operator. Two brackets were bonded to each tooth and were positioned at the central portion of the buccal surfaces, at 5 mm apart. Each bracket was light cured for 40 seconds (Poly Wireless; KaVo, Joinville, SC, Brazil) at an intensity of 1100 mW/cm2. The specimens were then stored in distilled water at 37ºC until the mechanical tests were performed.

**Table 1:**
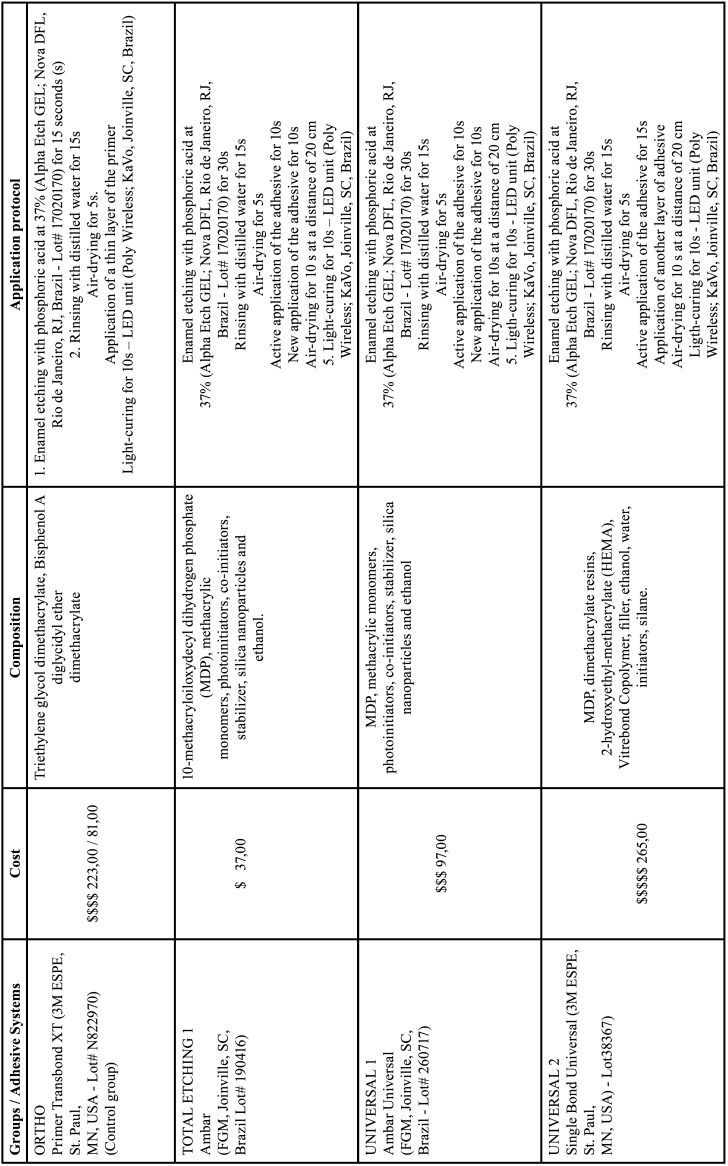
Tested adhesive systems, composition, and application protocols.

**Table 1 cont.**
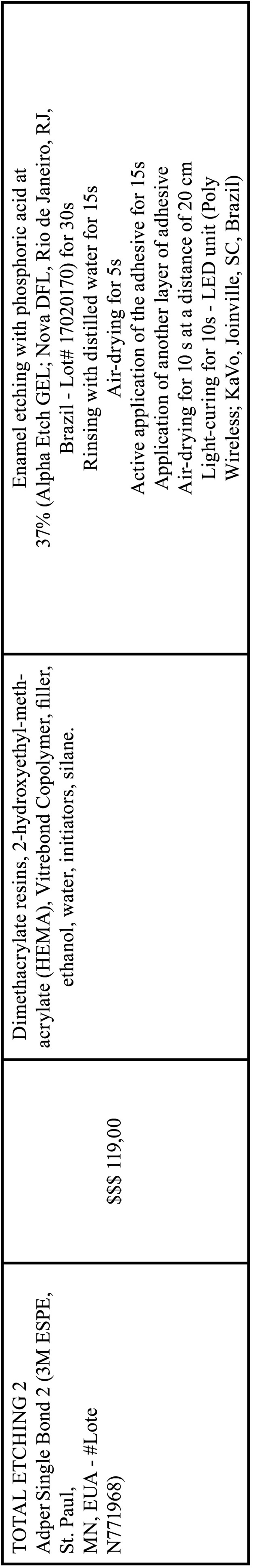
Tested adhesive systems, composition, and application protocols.

One bracket from each specimen was subjected to SBS test using a universal testing machine (EMIC 23-10, Instron Inc., Canton, MA, USA), at a speed of 0.5 mm/min, after a 24-hour storage period, while the others were evaluated after 12 months. The storage solution was changed biweekly to avoid bacterial proliferation and underwent pH monitoring (Phmetro, QUIMIS, Diadema, SP, Brazil). The maximum Force (F) applied for bracket detachment was recorded in Newtons (N), and the shear strength (ST) was calculated using the formula ST = F/A, where A corresponds to the base area of the bracket (10.5 mm2), providing values in Megapascal (MPa).

After bracket detachment, the enamel surfaces were analyzed using a stereo microscope (Stemi 305, Zeiss, Konigsallee, Germany), amplified 60X, to evaluate the amount of remaining adhesive, through the adhesive remnant index (ARI). The ARI assessments were performed by a single calibrated operator, obeying the scale idealized by Artun and Bergland(16), where the scores range from 0 to 3.

0: 0% of adhesive/composite left on the enamel, 100% on the bracket.

1: less than 50% of the adhesive/composite left on the enamel, more than 50% on the bracket.

2: more than 50% of the adhesive/composite left on the enamel, less than 50% on the bracket.

3: 100% of the adhesive/composite left on the enamel, 0% on the bracket.

Statistical Analysis 

The statistical analysis was performed using the Sigmastat 3.5 program (Systat Software Inc., San Jose, CA, USA). Initially, the Shapiro-Wilk normality test revealed a data distribution pattern outside the normality curve (*p*<0.05) for the bond strength and ARI values. Therefore, the bond strength values (M ± SD) were submitted to the Kruskal-Wallis test, and differences between groups were analyzed using the Student-Newman-Keuls post-test. Meanwhile, the ARI values, expressed as absolute frequency, were submitted to the Chi-square test. In all situations, a significance level of 5% was adopted.

## Results

-Bond strength 

The SBS values obtained after 24 hours, and 12 months are shown in Table 2. The SBS values were significant influenced by the adhesive system (*p*<0.001) and storage period (*p*<0.001), although no significant interaction was found between the latter two parameters (*p*=0.749).

**Table 2:**
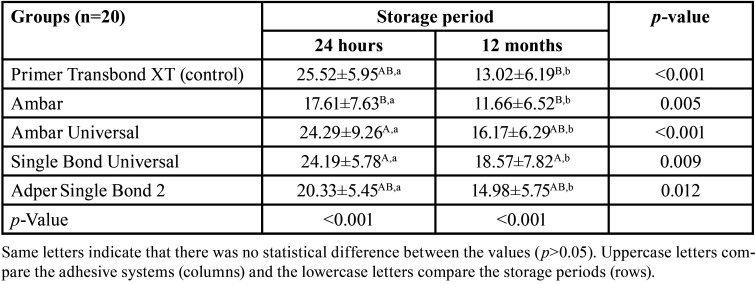
Bond strength values (MPa ± SD) according to the adhesive system and storage period.

After 24 hours, no significant difference in the SBS values was observed between the groups Primer Transbond XT, Ambar Universal, Single Bond Universal, and Adper Single Bond 2 (*p*>0.05). There was also no statistical difference between the AMBAR, Primer Transbond XT and Adper Single Bond 2 (*p*>0.05) groups. Nonetheless, a significant discrepancy was found between the Ambar group and the Ambar UNIVERSAL (*p*=0.015) and SINGLE BOND UNIVERSAL groups (*p*=0.011).

Meanwhile, after 12 months, no statistical difference was found between the SBS values of the groups Primer Transbond XT, Ambar, Ambar Universal, and ADPER Single Bond 2 (*p*>0.05). However, the Single Bond Universal group presented a statistically superior result when compared to the Primer Transbond XT (*p*=0.046) and AMBAR (*p*=0.011) groups.

When evaluating the preservation of bond strength over time, it was possible to observe that all groups significantly reduced the SBS values when comparing the 24-hour and 12-month storage periods (*p*<0.05).

-Adhesive Remnant Index (ARI) 

The distribution of the ARI is shown in Table 3. No statistical difference between the scores in each of the individually assessed groups (*p*>0.05) was observed, in the two evaluation periods. In turn, after 24 hours, a significant statistical difference was verified between the tested groups (*p*=0.043). The Primer Transbond XT and Ambar Universal adhesive system presented a statistically higher score 2 frequency than the other groups, while the Ambar, Single Bond Universal, and Adper Single Bond 2 groups showed a higher score 3 frequency than the Primer Transbond XT and Ambar Universal group. After 12 months, no statistical difference was observed in the ARI values between the analyzed groups (*p*=0.109).

**Table 3:**
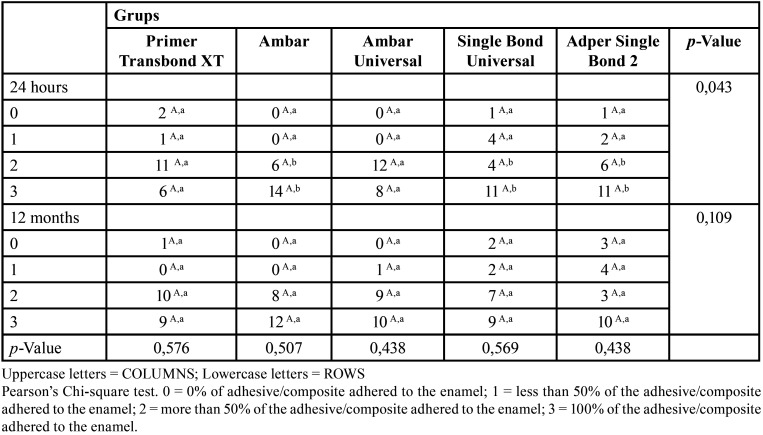
ARI values (absolute frequency) according to the adhesive system and storage period.

## Discussion

The present study showed the null hypothesis was rejected. The tested adhesive system, Ambar Universal and Single Bond Universal, have shown at least equivalent results to the gold standard Primer Transbond Universal XT adhesive in terms of SBD and ARI. In fact, Single Bond Universal consistently demonstrated better results, both in short- and long-term evaluations. These findings are in line with previous studies that have reported comparable or improved bond strength values with the use of alternative adhesive system (17).

Although some authors showed inferior (3,4,18) or no statistical difference results (2,12,19) when comparing universal adhesives with conventional ones, this may be justified by the absence of prior acid conditioning. The results obtained herein, with the superiority of Universal adhesives compared to conventional adhesives, can be justified by the acid etching prior to the application of these systems. Phosphoric acid etching causes the dissolution of the interprismatic enamel, generating a more irregular surface, which enhance the bond strength of the adhesive system and the orthodontic accessory (12,17,20). Therefore, adding the acid etching step to universal adhesive system improve their SBS results, and may be perform when superior bond is desired.

After 12 months of storage, the Single Bond Universal adhesive system retained satisfactory performance, with SBS values superior to the Ambar group and the Primer Transbond XT group, considered the gold standard for bonding orthodontic brackets (3). The superiority of Single Bond Universal may be attributed to the presence of 10-methacryloyloxydecyl dihydrogen phosphate (MDP) in its composition, which promotes chemical bonding with hydroxyapatite and contributes to the stability of the enamel interface/adhesive system (21). MDP is a molecule that promotes chemical bonding with the calcium ions of hydroxyapatite and is a predominantly hydrophobic monomer, a positive factor in terms of longevity, water sorption, and hydrolytic degradation over time (22). The addition of MDP to universal adhesive systems is a viable strategy to decrease the hydrolysis process, and consequently improve the durability of enamel binding (21). The Single Bond Universal adhesive system is composed of MDP, silica and silane, an association that may have led to an increase in the stability of the enamel interface/adhesive system. Therefore, Single Bond Universal adhesive (with previous acid etching step) seems to be a promising alternative for the bonding of orthodontic brackets. This is interesting due to cost involved with it, also, because the possibility to use the same adhesive system for restorations and bracket bonding facilitates everyday life at dental offices.

In studies that also compare the bond strength of different adhesive systems associated with Transbond XT orthodontic adhesive paste (11,12,14,19) no statistical difference between the groups evaluated were exhibited. It concluded that other adhesive systems can be used for bonding orthodontic brackets to enamel, corroborating our findings.

When evaluating the preservation of bond strength after 24 hours and 12 months, a significant reduction in value was verified in all groups, a fact already reported by other authors (23,24). Some methods can be used in laboratory to assess the longevity of bonding interfaces, such as the storage of specimens for long periods in distilled water (6,7,13). Specimen aging is fundamental for experiments in the laboratory environment to approach clinical reality. Although adhesive systems are sensitive to mechanical and thermal fatigue, the main factor affecting bond longevity is the hydrolysis suffered by the components of the bonding interface (23). In contrast with our results, Behnaz *et al*. (6) and Youssefinia and Mortezai (7) reported no decrease in bond strength after aging in distilled water. However, the maximum storage period in these studies was 3 months and 1 week, respectively, which may not be sufficient to cause significant alterations. Some may think that artificial saliva solutions can be used to mimic the clinical situation more accurately. Nonetheless, storage in water produces similar degradation results to artificial saliva, in addition to constituting a simple and low-cost solution (24).

In Dentistry, most studies evaluating the longevity of bonding interfaces focus on dentin adhesion, since they consider the bonding to enamel as predictable and stable. However, the enamel bonding interfaces is a crucial factor to consider in orthodontic treatments. Indeed, it has been well established that durable enamel bonds can be produced by the acid-etching technique followed by the application of primer and hydrophobic adhesive (23). On the other hand, the tendency of adhesive dentistry is to seek simpler solutions (25). After 12 months indicates the need for continuous monitoring and maintenance of orthodontic brackets. The hydrolysis process and the degradation of adhesive components over time are the main factors affecting the longevity of bonding interfaces (17). The addition of MDP to universal adhesive systems has shown promise in decreasing the hydrolysis process and improving the durability of enamel bonding (21).

According to Reynolds, the minimum accepTable bond strength for bonding orthodontic accessories to enamel ranges from 5.9 to 7.8 MPa. The mean bond strength values obtained by all the adhesive systems tested in the present study in the two evaluation periods exceed the minimum value, suggesting that all are suitable for bonding orthodontic accessories to the enamel, corroborating with the results reported by other previously mentioned authors (2,12,14,19). In contrast with restorative treatment, excessively high bond strength values are undesirable in Orthodontics, since they may increase the risk of enamel damage at the moment of bracket removal (6). Adhesive systems tested herein present a low risk of causing damage to the enamel, since there was a gradual reduction in bond strength values after 12 months. In addition, damage to the enamel was not observed during the ARI assessments in all groups.

The determination of the ARI scores provides insights into the quality of bonding at the adhesive/enamel and adhesive/bracket interfaces. When analyzing the obtained ARI results (Table 3), it noted a significant presence of scores 2 and 3. However, the Chi-square test did not reveal a statistical preference for any of these scores in each individually assessed group, both in the immediate analysis and after 12 months, a fact that may be justified by the reduced size of our sample. Thus, it can be suggested that the ARI distribution did not follow a specific pattern. Some studies (7,8,14) reported a predominance of samples with scores 2 and 3, indicating that most of the failures occurred in the adhesive/bracket bond interface. This type of failure may be considered preferable since adhesive/enamel interface failures are more likely to cause damage to the enamel surface at the time of accessory detachment (2,8,26). However, some authors disagree with this statement (5,15,27). In the study by Attar *et al*. (27), score 1 was dominant among the samples, whereas Hellak *et al*. (19) obtained a higher percentage of scores 0. These authors defend that scores 0 and 1 are desirable since there will be little adhesive to be removed from the enamel after detachment, decreasing chair time and the chance of scratching the enamel surface. Considering that current drills and polishing systems can remove the remaining adhesive without causing significant damage to the enamel, we believe that high ARI scores (2 and 3) are preferable.

In turn, we verified that after 24 hours the ARI values were influenced by the adhesive systems, revealing a statistical difference between the groups regarding the frequency of scores 2 and 3, where Ambar and Ambar Universal presented higher frequency for score 2 and 3 than the other adhesives tested. After 12 months, a statistical difference was not observed between the groups, in any of the evaluated scores. Divergent results were found in the literature concerning the ARI. Some studies (2,12) reported a significant difference in ARI values between the tested groups, while others (14,19) did not. ARI scores, which are not affected by bond strength values alone, depend on several factors, including bracket base design and type of adhesive. Therefore, it is noteworthy that the ARI scores are not always directly related to bond strength values (28).

Although the laboratory studies presented in this discussion provide valuable insights into the performance of adhesive systems, it is important to acknowledge their limitations. Some studies reveal significant differences between the bond strength values obtained in clinical and laboratory conditions (29,30), suggesting that *in vitro* test results cannot always be extrapolated to a clinical context, thus evidencing a limitation of the present study. In vitro studies do not always accurately reflect clinical conditions, and there may be variations between laboratory and *in vivo* results. Future research should focus on conducting clinical studies to validate the findings of laboratory studies and assess the long-term performance of adhesive systems in real-world orthodontic treatments.

## Conclusions

Within the limitations of the present study, alternative adhesive systems, such as Ambar Universal and Single Bond Universal are efficient in bonding orthodontic brackets to enamel when associated with Transbond XT adhesive paste. Moreover, these alternative adhesive systems offer practical advantages, including cost-effectiveness and versatility for other dental procedures. The use of an etch-and-rinse strategy with universal adhesives enhances their bond strength on enamel. However, the long-term durability of bonding interfaces remains a challenge, and continuous monitoring and maintenance are necessary. Future research should focus on conducting clinical studies to validate the laboratory findings and assess the performance of adhesive systems in real-world orthodontic treatments.
